# Identification of genes differentially expressed between benign and osteopontin transformed rat mammary epithelial cells

**DOI:** 10.1186/1756-0500-2-15

**Published:** 2009-02-03

**Authors:** Vittal V Kurisetty, Patrick G Johnston, Philip S Rudland, Mohamed K El-Tanani

**Affiliations:** 1 Centre for Cancer Research and Cell Biology (CCRCB), Queen's University Belfast, Belfast, BT9 7BL, UK; 2 Cancer and Polio Research Fund Laboratories, School of Biological Sciences, University of Liverpool, PO. Box 147, Liverpool, L69 7ZB, UK

## Abstract

**Background:**

Osteopontin is a secreted, integrin-binding and phosphorylated acidic glycoprotein which has an important role in tumor progression.

**Findings:**

In this study, we have utilized suppressive subtractive hybridization (SSH) to evaluate OPN regulated gene expression, using the Rama 37 benign non-invasive rat mammary cell line and a subclone, Rama 37-OPN. Rama 37-OPN was produced by stably transfecting Rama 37 with an OPN expression vector and it demonstrates increased malignant properties *in vitro*. Sequence and expression array analysis of the respective cDNA libraries of over 1600 subtracted cDNA fragments revealed 982 ESTs, 45 novel sequences and 659 known genes. The known up-regulated genes in the Rama 37-OPN library code for proteins with a variety of functions including those involved in metabolism, cell adhesion and migration, signal transduction and in apoptosis. Four of the most differentially expressed genes between the benign and *in vitro *malignant rat mammary cell lines are tumor protein translationally controlled I (TPTI), aryl hydrocarbon receptor nuclear translocator (ARNT), ataxia telangiectasia mutated (ATM) and RAN GTPase (RAN). The largest difference (ca 10,000 fold) between the less aggressively (MCF-7, ZR-75) and more aggressively malignant (MDA MB 231, MDA MB 435S) human breast cancer cell lines is that due to RAN, the next is that due to osteopontin itself.

**Conclusion:**

The results suggest that enhanced properties associated with the malignant state *in vitro *induced by osteopontin may be due to, in part, overexpression of RAN GTPase and these biological results are the subject of a subsequent publication [1].

## Background

Metastasis is the major cause of treatment failure in breast cancer patients [1]. The extracellular matrix glycophosphoprotein osteopontin (OPN) is normally secreted by osteoblasts and is utilized as an extracellular adhesion molecule [1]. Osteopontin has also been associated with certain aspects of malignant transformation [2] by enhancing malignant cell attachment contributing to anchorage-independent growth and the migration of tumor cells [3,4]. Moreover, transfection of benign, nonmetastatic rat mammary cells with the cDNA for OPN in an expression vector endows the transfectants with the ability to overproduce OPN *in vitro *and to metastasize *in vivo *[5]. Furthermore, we and others have shown that OPN overexpression is associated with poor prognosis in human primary breast cancer [6,7]. Recently, OPN has been shown to be the single most powerful prognostic factor in a multivariate analysis against outcome, in a large prospective study of breast cancer patients [8]. Circulating plasma levels of OPN are also higher in metastatic breast cancer patients [9]. However, the precise molecular mechanisms of how OPN regulates metastasis remain unclear.

To obtain a better understanding of this phenomenon and, in particular, the downstream target genes in the OPN signaling pathway, the technique of suppression subtractive hybridization (SSH) has been employed. SSH is an efficient method that uses widely available facilities to identify sequences of known and unknown genes [10]. SSH has been used in the present study to determine differential gene expression between the benign rat mammary cell line Rama 37 (R37) and R37 cells stably transfected with an expression vector for OPN, termed R37-OPN cells.

Using the SSH method combined with reverse Northern hybridization, we have identified genes that are differentially expressed between the benign non-invasive rat mammary cell line and the invasive and malignant metastatic Rama 37-OPN. Some of the differentially expressed genes were then tested further by Real Time PCR for the relative level of their expressed mRNAs in a series of cell lines established from human breast cancer and the mRNA species which changes the most has been identified as RAN GTPase. The biological relevance of these changes are the subject of a subsequent paper [1].

## Results

### Stable transfection of osteopontin gene

**Figure 1:**
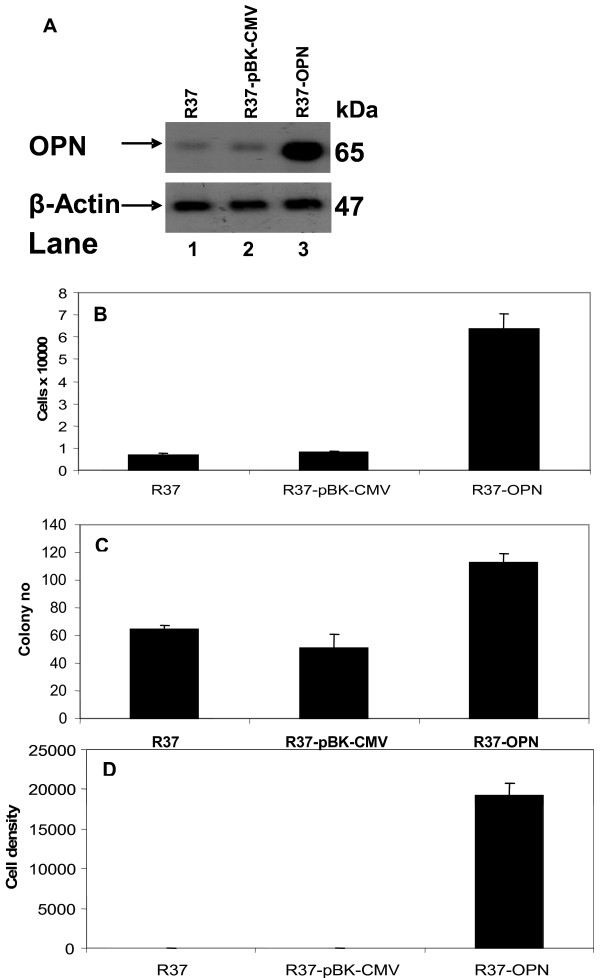
**The biological effect of introduction of OPN into benign R37 cells**. ***A ***Immunoblot showing OPN protein levels in R37, R37-pBK-CMV and R37-OPN cells. Cell lysates were diluted and 20 μg loaded onto a SDS 10% (w/w) polyacrylamide gel as follows: *lane 1 *R37; *lane 2; *R37-pBK-CMV; *lane 3 *R37-OPN. Specific proteins were detected using antibodies to OPN and β-actin. Bands were quantified using densitometric analysis and normalized against β-actin. The average fold increase for three different experiments are: lane 1 = 1, lane 2 = 1 ± 0.2 and lane 3 = 10 ± 1.7. ***B ***Ability of transfected cell lines to adhere to a fibronectin-treated surface was assessed over a 30 min period and the number of adherent cells quantified. Results of the mean ± standard error from three independent experiments are shown. ***C ***Soft agar assay was carried out to assess the ability of stably transfected cell lines to grow in an anchorage independent environment. The colony number was assessed after 5 days. Results of the mean ± standard error from three independent experiments are shown. ***D ***R37, R37-pBK-CMV and R37-OPN were plated on ECM-coated filters (500 μg/ml) in Boyden chambers. The number of cells that migrated through the filter after 48 hrs was determined by staining and scanning using a digital imaging system (Methods). Results of the mean ± standard error from three independent experiments are shown.

To ascertain if OPN expression was related to invasion and other properties associated with the malignant state *in vitro*, we raised various stable cell lines from R37 cells. These were stably transfected by empty vector pBK-CMV (R37-pBK-CMV) or by the constitutively-active expression vector OPN-pBK-CMV (R37-OPN cells), as described in Methods. Individual clones of the transfectants were combined for subsequent analysis to generate pooled cell lines. Immunoblot analysis using a monoclonal (MAb) antibody to OPN, which recognizes both human and rat OPN (Materials and Methods), showed that the OPN protein was expressed at a low level in R37 and R37-pBK-CMV cells (Fig. 1A). OPN protein expression was increased 10 fold in R37-OPN compared to R37 and R37-pBK-CMV cell lines (Fig. 1A). In R37, R37-pBK-CMV and R37-OPN cell lines, the MAb to OPN recognized a protein of *M*_r _65,000 (Fig. 1A, *Lanes 1–3*), consistent with the size of OPN from the original rat cell lines [8,11]. The increase in OPN protein in the R37-OPN pooled cell line suggests that the OPN-pBK-CMV vector consistently overexpresses OPN, the empty vector pBK-CMV having no effect on OPN expression in R37 cells.

### Effect of OPN on cellular adhesion, anchorage-independent growth and invasion

Adhesion of cells to fibronectin-coated surfaces was assayed using a dye-based system. R37-OPN cells showed a 9.2 and 8 fold increase in cell adhesion to fibronectin-coated dishes, in comparison with R37 and R37-pBK-CMV cells, respectively (Student's t-test, p < 0.01) (Fig. 1B). Colony formation was assayed in soft agar, R37-OPN cells induced a 1.8 and 2.3 fold increase in colony number per plate compared to R37 and R37-pBK-CMV cells, respectively (p < 0.01) (Fig. 1C). The ability of cells to migrate through a reconstituted three dimensional collagen gel (Matrigel) and appear on the underside of a polycarbonate membrane was tested as an assay for cell invasion. The cells on the underside of the membrane were stained, scanned and counted using a digital imaging system described in Methods [12]. Migration of R37-OPN transformants was 913 and 1006 fold greater than the parental R37 and R37-pBK-CMV cells, respectively (p ≤ 0.002) (Fig. 1D). These results suggest that high levels of OPN induce cell adhesion, anchorage-independent growth and invasion *in vitro*, properties consistent with the malignant metastatic state *in vivo *[5,13].

### cDNA library construction by suppression subtractive hybridization

A cDNA library was constructed from the two cell lines. In the forward SSH library, the benign noninvasive R37 was used as the driver and the malignant invasive R37-OPN cell line as the tester. In the reverse suppression subtractive hybridization library, the benign, noninvasive R37 was used as the tester and the malignant invasive R37-OPN cell line as the driver. The forward and reverse subtracted libraries represent genes that are preferentially up and down-regulated in relation to OPN-mediated transformation in mammary cells. To evaluate the efficiency of cDNA subtraction, we compared the transcript levels of the housekeeping gene glyceraldehyde-3-phosphate dehydrogenase (GAPDH) by RT-PCR in subtracted and unsubtracted cDNA libraries from R37 and R37-OPN cells, respectively. Detection of GAPDH sequences for both subtractions required 26 PCR cycles with subtracted cDNA as template, whereas only 10 cycles were required to amplify GAPDH from control cDNAs. Thus the commonly expressed gene GAPDH was significantly depleted from the subtracted cDNA libraries. Overall, 90% of the randomly-picked cloned cDNAs yielded sequence information. We have obtained 291 sequences (comprising 108 different known cDNAs (see Additional file 1), 178 expressed-sequence-tags (ESTs) (see Additional file 2) and 5 unmatched sequences) and 1394 sequences (comprising 550 different known cDNAs (see Additional file 3), 804 expressed-sequence-tags (ESTs) (see Additional file 4) and 40 unmatched sequences) from R37 and R37-OPN subtracted libraries, respectively. In total, 327 subtractive cDNA clones (303 in the R37-OPN and 24 in the R37), after excluding redundant and false-positives, were obtained by performing a BLAST (Basic Local Alignment Search Tool) search for comparison with NCBI RefSeq, GenBank, and dbEST. The known and novel genes in R37 (see Additional files 1 &2) and R37-OPN (see Additional files 3 &4) libraries are listed according to the function of their proteins. Sequence analysis of the colonies from R37-OPN and R37 libraries yielded a complex of previously identified cancer-associated genes, tumor suppressor genes as well as a set of previously uncharacterized genes with no level of redundancy. These data are consistent with a significant enrichment for invasion-associated and suppressor genes from the R37-OPN and R37 libraries, respectively.

### Differential screening of the subtracted libraries

The relative levels of mRNAs corresponding to the sequenced cloned cDNAs, isolated from the subtracted benign, non malignant and invasive, malignant libraries, were estimated in R37 and R37-OPN cells by reverse Northern hybridization, using cDNA produced from mRNA from either the R37 or R37-OPN cells as probes. The hybridization results were normalized using cDNAs corresponding to mRNAs for GAPDH, which showed similar expression between the R37 and R37-OPN cell lines. Using an expression ratio of over three-fold as cut-off, 18 of 24 cDNA clones and 83 of the 303 cDNA clones, examined by reverse Northern screening were identified in the benign, non-invasive and malignant, invasive subtracted libraries, respectively (see Additional files 1 &2 and 3 &4). Eighteen % and 38% cDNAs were differentially expressed by over 15-fold in the benign and malignant breast tumor cell lines, respectively. Amongst the cDNAs expressed at a higher level in the R37-OPN cells than in the R37 cells were previously characterized breast cancer-associated genes such as TPTI [14], ARNT [15], CSFIR [16], MDM2 [17], CD44 [18], Cxcr4 [19], RAN GTPase [20], cytokeratin 20 (CK20) [21], Ki67 [22], with fold increases of 8, 8, 9, 9, 11, 23, 25, 32, and 45, respectively. Amongst the cDNAs expressed at a lower level in R37-OPN cells were previously characterized cancer suppressor genes PTEN [23], ATM [24] and BRCA1 associated protein 1 [24] with decreases of 19, 23 and 23 fold, respectively. These results are consistent with changed levels of expression of molecules implicated in breast and other cancers.

### Quantitative Real Time PCR

Four of the highest differentially expressed genes that were also associated with invasion and/or malignancy were chosen from the total number of 1685 for validation by quantitative real-time PCR analysis (QPCR) (see Additional file 5) using the relatively non-invasive MCF-7 and the highly invasive MDA-MB-231 and MDA-MB-435S human breast cancer cell lines [25]. The four genes encode for tumor protein translationally-controlled (TPTI), aryl hydrocarbon receptor nuclear translocator (ARNT), ataxia telangiectasia mutated (ATM) and RAN GTPase (RAN). MCF-7 cell: other cell ratios were calculated using the comparative threshold cycle (Ct) method [26] after normalization to a control housekeeping gene for ribosomal RNA S18. The highly invasive malignant cell lines MDA-MB-231 and MDA-MB-435S showed a 269 and 159 fold increase in TPTI expression, respectively, in comparison with the relatively non-invasive malignant cell line MCF-7. MDA-MB-231 and MDA-MB-435S cells showed a 16.6 and 4.1 fold increase in ARNT expression, respectively, in comparison with MCF-7 cells. ATM was undetectable in both MCF-7 and MDA-MB-435S, but a small amount was detected in MDA-MB-231 cells (see Additional file 5). However, the most differentially expressed gene of the four was RAN GTPase. Thus the invasive cell lines MDA-MD-435s and MDA-MB-231 showed a 13191 and 765 fold increase, respectively, in RAN expression compared to MCF-7 cells and they also showed a 9785 and 60 fold increase in levels of OPN (see Additional file 5). The relatively non-invasive breast cancer cell line ZR-75 showed almost identical levels of low expression of RAN and OPN to those of the MCF-7 cell line (see Additional file 5).

## Discussion

These results are not a simple artefact of a single clone of cells, since they have been obtained with pools of single cell clones of transfectants. R37 cells thus provide a robust system for the study of OPN-induced changes in the parental R37 cells that lead to the invasive, malignant phenotype *in vitro *and, in a parallel system, to the metastatic state *in vivo *[5]. Using SSH technology, we have now identified potential downstream effectors of the OPN-induced signaling network that may lead to invasion and ultimately metastasis. Here we report the recovery of gene fragments representing differentially expressed mRNAs between benign, noninvasive R37 [11] and malignant, invasive R37-OPN cells [11] from two cDNA libraries established after SSH, and compare their sequences with those of known genes (see Additional files 1 &2, 3 &4). The SSH procedures used to establish differences in gene expression between the two cell types have permitted the isolation of genes expressed in high and low-abundance classes. It also leads directly to the production of cloned fragments of expressed genes without the necessity to clone subsequently genes of interest.

## Methods

### Cell culture and production of stable transformant cell lines

#### *In vitro *tests for malignancy

Assays for cell adhesion, colony formation and invasion through Matrigel in Boyden chambers were carried out as previously described ([13]; see Additional file 6).

#### Synthesis of SSH cDNA libraries

Two subtracted cDNA libraries, from R37 and R37-OPN cell lines, were synthesized using the PCR-Select™ cDNA subtraction kit (CLONTECH) (see Additional file 6).

#### Cloning and sequence analysis of OPN-target genes

The forward and reverse subtracted cDNAs were cloned into pCR2.1-TOPO vectors (Invitrogen) and transformed into competent TOPO 10 cells (Invitrogen) (see Additional file 6).

#### Expression array screening and analysis

A total of 327 combined cDNA inserts from forward subtracted and reverse subtracted libraries were PCR amplified using NP1R and NP2R primers as previously described [27] (see Additional file 6).

#### Quantitative Real Time RT-PCR (QPCR)

QPCR was used as an independent method to probe the association of identified differentially expressed genes with that of OPN in different human breast cancer cell lines (see Additional file 6).

#### Statistical treatment of results

All biological experiments were performed at least 3 times. The mean and standard error were calculated and p values less than 0.05 were considered significant as calculated using the Student's t-test.

## The abbreviations used are

OPN: osteopontin, MAb: monoclonal antibody; Rama: rat mammary; and SDS: sodium dodecyl sulfate.

## Authors' contributions

VVK: Carried out the laboratory molecular biology studies and sequence alignment; PGJ: Participated in data analysis and revising the manuscript; PSR: Provided the parental Rama 37 and revising the manuscript and MKE: conceived, coordinated and designed the study, led the research, data analysis and wrote the entire manuscript. All the authors read and approved the final manuscript.

## Supplementary Material

Additional File 1 **Table 1A.** Known Genes from R37-OPN Subtracted Library. Species name and accession number are listed according to the match in BLAST analysis http://www.ncbi.nlm.nih.gov/blast/blast.cgi. Click here for file

Additional File 2 **Table 1B.** ESTs from R37-OPN Subtracted Library. Click here for file

Additional File 3 **Table 2A Known Genes from R37 Subtracted Library**. Click here for file

Additional File 4 **Table 2B.** ESTs from R37 Subtracted Library. Click here for file

Additional File 5 **Table 3. **Quantitative RT-PCR for 3 breast cancer cell lines. The differential expression of the tumor protein, translationally-controlled 1 (TPT1), aryl hydrocarbon receptor nuclear translocator (ARNT), ataxia telangiectasia mutated (ATM), and RAN GTPase (RAN) genes versus the housekeeping S18 ribosomal gene was calculated for each cell line using the expression: &#916Ct = Cttarget gene-CtS18. Next, MCF7 non-invasive cells:other cell ratios were calculated from the &#916Ct values as follows: 2-(&#916Ct tumor cells-&#916Ct MCF-7). ND-not detectable. Click here for file

Additional File 6 **Details Methods**. Click here for file
