# Supplementary material for: Identification of genes differentially expressed between benign and osteopontin transformed rat mammary epithelial cells
Source: BMC Res Notes. 2009 Feb 3;2:15. doi: 10.1186/1756-0500-2-15 (PMC2644310; doi:10.1186/1756-0500-2-15)
Supplement: Additional File 6 — Details Methods. [file 1756-0500-2-15-S6.pdf]

Details Materials and Methods

Cell lines and cell culture. The rat mammary (R37) nonmetastatic benign tumor-derived cell line described previously [12] and derivative stably transformed cell subclones were cultured in Dulbecco's modified Eagle's medium (DMEM), 10% (v/v) fetal calf serum, 100 µg/ml penicillin, 100 µg/ml streptomycin (Invitrogen). The MCF-7 human breast cancer cell line was obtained from ECACC, Wiltshire (UK) and propagated in DMEM, 5% ($v\ v^{-1}$) FCS, 50 ng ml^{-1} insulin, 10^{-8} M estradiol. The human breast cancer cell lines ZR-75, MDA-MB 231 and MDA-MB 435S, were obtained from ECACC, Wiltshire (UK), and were grown in RPMI supplemented with 20% (v/v) fetal calf serum,1 mM sodium pyruvate, and 100 µg/ml penicillin-streptomycin.

Production of stable transformant cell lines. R37 and its derivative cell line were cultured as outlined above. Cells were harvested and seeded in multiwell plates at 2.5 $x10^5$/3.5cm diameter well in 1ml of serum-free medium. Initially R37 cells were transfected with an expression vector for OPN as previously described using 1.0 mg/ml geneticin (Invitrogen) to yield single cell clones of transfectants [14]. The surviving cell colonies following transfection and growth under selective pressure were combined to yield the pooled cell line R37-OPN.

Western blotting for OPN protein. OPN protein levels were established in the stably transfected cells by Western blotting as described previously [50]. Total protein of 20 µg and 5 µg respectively, from whole cell lysates of OPN transfected cells were electrophoresed through 10% (w/v) polyacrylamide, 1% (w/v) SDS gels and transferred by blotting onto nitrocellulose membranes (Millipore Corporation, Watford, U.K.). The

membranes were blocked by incubation with 0.02 M Tris-HCl (pH 7.0), 0.9% (w/v) NaCl, 0.1% (v/v) Tween 20 containing 5% (w/v) Marvel, for 1 hour. Monoclonal antibodies to OPN (1/500) (Developmental Studies Hybridoma Bank, Iowa City, IA) was added overnight at 4°C. Bound antibodies were located by a further incubation with 1:5000 horseradish peroxidase-conjugated rabbit antimouse Ig, visualized with Luminol Reagent (Santa Cruz Biotechnology, Inc, California, USA) and exposed to Kodak XAR5 film (Sigma, Poole, U.K.). Bands on films were quantified using a digital imaging system (Syngene, Genetool, Cambridge, England).

In vitro tests for cell adhesion. Cell adhesion assays were carried out as previously described [51]. Briefly cells were plated at a known density (2 x 10^5 cells per well) in a 6 well plate in conditioned media and allowed to adhere to fibronectin-coated plates for 30 min at 37°C, in an atmosphere of 5% (v/v) CO_2. Cells were then washed with PBS and, after their removal by treatment with tryspin/EDTA, were counted and cell adhesion expressed as a percentage of those adhered compared to the total number of cells added.

In vitro tests for colony formation. Assays were carried out as described previously [15]. For the bottom agar, 5 ml of 1.6% (w/v) agarose was plated in a 100-mm diameter tissue culture dish and allowed to harden. Cells were removed by trypsinization and resuspended at 1.0 x 10^6 cells/ml in normal medium. Nine ml of normal media (1x DMEM with 10% (v/v) FCS, 200mM L-glutamine, 100 μg/ml penicillin, 100 μg/ml streptomycin) was added to the top of the bottom agar and 1.0 ml of cells seeded per plate. The plates were incubated at 37°C in an atmosphere of 5% (v/v) CO_2 for 5-7 days

and stained with 1ml of 0.2 % (w/v) crystal violet. The plates were scanned for colonies and counted using a digital imaging system (Syngene, Genetool, Cambridge, England) [15].

Invasion through matrigel measured in Boyden chambers. Biocoat 250 μg/ml Matrigel invasion chambers 6.4 mm in diameter (Falcon-Ulster Anaesthetics, Moneyrea, NI, UK) were used to assess the invasiveness of R37 and R37-OPN cells, as described previously [15] . Briefly, $1x10^6$ cells were resuspended in 1ml of serum-free DMEM and 100 μl added to the cell culture inserts of the upper invasion chambers on top of Biocoat 250μg/ml Matrigel-coated invasion chamber. A chemoattractant, 5μg of rat fibronectin (Gibco-BRL, U.K.) per ml in DMEM and 10% (v/v) FCS were added to the lower chambers. The cultures were incubated at 37°C in a 5% (v/v) CO_2 atmosphere and allowed to invade through the matrix and the pores (8μm) of the attached lower membrane for 48h. The upper surfaces of the filters were wiped clean of cells and the filters were fixed by immersion in 100% (v/v) methanol and stained by Gurr's eosin and methylene blue, according to the manufacturer's instructions (BDH Laboratory Supplies, Pool, U.K). The inserts were scanned for cell density using a digital imaging system (Syngene, Genetool, Cambridge, England) [15].

mRNA Isolation. Cells were harvested at an exponential phase for RNA isolation. Total RNA was isolated with TriZol reagent (Life Technologies, Paisley, Scotland, UK) following the manufacturer's instructions. mRNA was isolated from total RNA with a NucleoTrap mRNA extraction kit (BD Clontech, Oxford, UK) following the manufacturer's protocol.

Synthesis of SSH cDNA libraries. Two subtracted cDNA libraries, from R37 and R37-OPN cell lines, were synthesized using the PCR-Select™ cDNA subtraction kit (CLONTECH). In a forward subtracted cDNA library, poly $(A)^+$ RNA was extracted from the R37-OPN cell line (as the tester) and from R37 parental cell line (as the driver) and was used to synthesize cDNA. Conversely a reverse subtracted cDNA library was constructed using poly $(A)^+$ RNA from the R37-OPN cell line as the driver and from the R37 parental cell line as the tester. Tester and driver cDNAs were synthesized from 2 µg poly $(A)^+$ RNA using the cDNA synthesis primer 5'–TTTTGTACAAGCTT$_{30}$N$_1$N–3' and AMV Reverse Transcriptase (CLONTECH). Rsa1 digested tester cDNA was ligated to adaptor 1 (1R) (5'CTAATACGACTCACTATAGGGCTCGAGCGGCCGCCCGGGC AGGT-3') and adaptor 2 (2R) (5'CTAATACGACTCACTATAGGGCAGCGTGGTCG CGGCCGAGGT-3') using T4 DNA ligase (CLONTECH). 0.45 µg driver cDNA was added to each of the two tubes containing equal amounts of adaptor-1-ligated tester and adaptor 2R-ligated tester. The reaction mixture was denatured at 98^0C for 1.5 min and then incubated at 68^0C for 9 h. The samples were then combined with excess heat-denatured driver cDNA (270 ng) and incubated at 68^0C overnight. Primary PCR was performed using PCR primer 1 (5'-CTAATACGACTCACTATAGGGC-3') with cycling conditions: 94^0C for 25 sec, 27 cycles at 94^0C for 10 sec, 66^0C for 30 sec, and 72^oC for 1.5 min. This was followed by secondary PCR and was performed using nested PCR primer NP1R (5'-TCGAGCGGCCGCCCGGGCAGGT–3') and nested PCR primer NP2R (5'–AGCGTGGTCGCGGCCGAGGT–3') with cycling conditions: 15 cycles of 94^0C for 10 sec, 68^0C for 30 sec, and 72^0C for 1.5 min. (MJ Research Thermal Cycler, Helena Biosciences, Sunderland, England).

Cloning and sequence analysis of OPN-target genes. The forward and reverse subtracted cDNAs were cloned into pCR2.1-TOPO vectors (Invitrogen) and transformed into competent TOPO 10 cells (Invitrogen). The above clones were screened by colony PCR in which the template DNA was replaced by a colony picked from the agar plate. DNA sequencing was performed using the ABI377 (Perkin Elmer/Applied Biosystems, Foster City, CA, USA), with universal M13F and M13R primers. DNA homology database searches were performed using the NCBI BLAST program. Overall library redundancy and complexity was evaluated based on the frequency of unique clones recovered. Highly redundant clones were used as probes to prescreen the libraries prior to the generation of arrays.

Expression array screening and analysis. A total of 327 combined cDNA inserts from forward subtracted and reverse subtracted libraries were PCR amplified using NP1R and NP2R primers with amplifying conditions: 94^0C for 36 sec; 25 cycles of 95^0C for 18 sec and 68^0C 3 mins (MJ Research Thermal Cycler, Helena Biosciences, Sunderland, England). Amplified clones were visualized using 2.0% agarose gels and stained with ethidium bromide. Dot blotting equipment was used to spot each purified PCR reaction onto a nylon membrane (Hybond-XL) by combining 2μl of PCR product and 2μl of 0.6M NaOH (freshly made). The blots were then neutralized by using 0.5 M TrisHCl (pH 7.5) and washed with sterile distilled H_20 followed by UV cross-linking of the DNA to the membrane using UV Stratalinker (Stratagene). Dot blots were hybridized with forward subtracted and forward unsubtracted cDNA (tester & driver) and reverse subtracted and

reverse unsubtracted probes (tester & driver) that were random-primed labeled with [α-^{32}P] dCTP (Amersham). Hybridizations were performed overnight at 72°C in 20x SSC (0.45 M NaCl, 45 mM sodium citrate, pH 7.0), purified probes and blocking solution (CLONTECH). Membranes were washed four times with low stringency wash solution (2x SSC/0.5% SDS) for 20 min each at 68^0C, followed twice with high stringency washes (0.2 x SSC/0.5% SDS) for 30 min each at 68^0C. PhosPhorImager SI (Molecular Dynamics, Sunnyvale, CA, USA) and Array Vision 6.0 software (Imaging Research, St. Catherine's, ON, CA, USA) was used to calculate the array differentials. The housekeeping control gene GAPDH was used to assess hybridization signal equivalence. The reproducibility of expression array screening was ensured by the inclusion of duplicate controls at various locations on the membrane. In addition, several previously described breast cancer genes were identified multiple times throughout the screening process.

Quantitative Real Time RT-PCR. Quantitative PCR (QPCR) was used as an independent method to probe the association of identified differentially expressed genes with that of OPN in different human breast cancer cell lines. QPCR analysis was performed using a QuantiTect SYBR® Green RT-PCR kit (Qiagen) containing a QuantiTect SYBR Green RT-PCR buffer, SYBR Green I dye and ROX passive reference dye. In comparing quantitative expression differentials, three different human cell lines were utilized. Each sample was run in triplicate to ensure quantitative accuracy. QPCR data are reported as a ratio of the non-invasive human breast cancer MCF-7 cells to the invasive MDA MB 231 or MDA MB 435S cells and calculated using the comparative

threshold cycle (Ct) method [28]. Briefly, the differential expression of different target genes versus the housekeeping gene, ribosomal RNA (S18), was first calculated for all samples using the expression: ΔCt=Ct of the target gene-Ct of the reference (S18). MCF-7 cells were compared to the indicated cell lines using the following formula: ΔΔCt = ΔCt value for target gene - ΔCt value for MCF-7 cells. Values were reported as $2^{-(\Delta\Delta Ct)}$ [28]. All reported QPCR reactions were performed and analyzed using a LightCycler rapid thermal cycler system (Roche, East Sussex, England).

Briefly, 1μg of total RNA was reverse transcribed with Superscript II reverse transcriptase (Invitrogen, Life Technologies Ltd, Paisley, UK). The cDNA was then diluted to give 200 μg/μl prior to PCR amplification. Reactions were performed in a 10 μl volume using 200 μg of cDNA and 5 μl QuantiTect SYBR Green PCR Master Mix (Qiagen, West Sussex, UK). Control samples without reverse transcriptase enzyme were included for each cell type. The expression of TPT1, ARNT, and ATM were quantified using the following primers: forward 5'-GATCGCGGACGGGTTGT-3', reverse 5'-TTCAGCGGAGGCATTTCC-3', forward 5'-GCTGCTGCCTACCCTAGTCTCA-3', reverse 5'-GCTGCTCGTGTCTGGAATTGT-3' and forward 5'-CAGGGTAGTTTAGTTGAGGTTGACAG-3', reverse 5'-CTATACTGGTGGTCAGTGCCAAAGT-3'. The expression of RAN was quantified using the following primers: forward 5'-TACTGGAAAAACGACCTT-3', reverse 5'-TCCCATACATTGAACTTA-3'. Internal control primers of ribosomal RNA (S18) were also included and were as follows: forward 5'-GTAACCCGTTGAACCCCATT-3', reverse 5'-CCATCCAATCGGTAGTAGCG-3'. The protocol included a 15 min activation step at 95°C, followed by 40 cycles of 15 sec denaturation at 94°C, 30 sec of

predetermined optimum annealing temperature and extension for 30 sec at 72^oC. An amplification plot of fluorescence signal versus cycle number was drawn. In the initial cycles of PCR there was little change in fluorescence signal and this defined the baseline for the amplification plot. An increase in fluorescence above the baseline indicated the detection of the accumulated PCR product. A fixed fluorescence threshold of 0.1 was set above the baseline in the exponential phase of the PCR. To confirm amplification specificity, the PCR products from each primer pair were subjected to a melting curve analysis and checked using agarose gel electrophoresis.
